# Potentially modifiable blood triglyceride levels by the control of conventional risk factors

**DOI:** 10.1186/s12944-019-1134-0

**Published:** 2019-12-13

**Authors:** Bumjo Oh, Joohon Sung, Sohyun Chun

**Affiliations:** 1 Department of Family Medicine, SMG-SNU Boramae Medical Center, Seoul, 07061 South Korea; 2 Department of Epidemiology, School of Public Health Seoul National University, Seoul, 08826 South Korea; 3 Institute of Health and Environment, Seoul National University, 1 Gwanak-ro, Gwanak-gu, Seoul, 08826 South Korea; 4 International Healthcare Center, Samsung Medical Center, 81 Irwon-ro, Gangnam-gu, Seoul, 06351 South Korea

**Keywords:** Triglyceride, Hyperlipidemia, Twin, Cohort, Metabolic syndrome

## Abstract

**Backgrounds:**

Triglyceride (TG) is known to be regulated by multiple lifestyle factors rather than genetic factors. This cross-sectional and community-based study (Healthy Twin study in Korea) aimed to estimate the “modifiable TG level” by identifying non-genetic risk factors of TG.

**Methods:**

Participants were recruited between 2006 and 2011 who fulfilled health examinations and detail surveys: 3079 Korean adults including 949 monozygotic twins and 222 dizygotic twins. In order to investigate conventional risk factors, a mixed model accounting for family as a random effect was performed. In addition, we conducted a co-twin control analysis for 452 monozygotic twin (MZ) pairs, to examine non-genetic risk factors and potentially modifiable serum triglyceride levels.

**Results:**

After excluding patients on dyslipidemia or diabetes medication, 2672 individuals (1029 men, with mean age of 43.9; and 1643 women with mean age of 43.3; 949 MZ pairs, 222 dizygotic twin pairs, and 1501sibling pairs) were analyzed. Fasting blood sugar (FBS), lipid panel, height, weight, waist (WC) and hip circumference, body mass index (BMI), amount of dietary intake and amount of physical activity was examined after adjusting for age and sex. For conventional analysis, WC, fat %, and BMI were identified as significant factors influencing serum triglyceride levels. Examination of non-genetic factors from the Co-twin control study revealed BMI (beta coefficient 9.94 with C.I. 3.42 to 16.46) and amount of alcohol intake (beta coefficient 0.08 with C.I. 0.02 to 0.14) as significant factors.

**Conclusion:**

Our findings suggest that controlling body weight and alcohol intake might be effective to control TG; moderate weight control (BMI 1 reduction) and reducing alcohol consumption by 50 g/week (about two glassed of beer) might reduce TG level by 9.94 and 4.0 mg/dL.

## Background

Recent changes in lifestyle and diet have led to changes in disease distribution [1]. Once, communicable diseases such as tuberculosis or pneumonia were the primary cause of death, but at the present time, aggressive neoplasm, cardiac diseases, vascular diseases of the brain, diseases related to hypertension, and diabetes account for over 70% of adult mortality [2]. In Korea in particular, the mortality caused by ischemic heart diseases (angina and acute myocardial infarction) is showing a rapid increase, growing from 2.2/100,000 persons in 1983 to 13.1/100,000 persons in 1995 and 27.5/100,000 persons in 2005 [3]. It has been reported that the risk factors for these coronary arterial heart diseases are, in men, hyperlipidemia, cigarette smoking, obesity, stress, hypertension, past history of stroke, and diabetes [4].

Hyperlipidemia in particular is a condition where excessive amounts of lipids accumulate in the walls of blood vessels, causing inflammation and ultimately coronary arterial heart diseases, in which increase in specific lipid levels comes as a result of genetic factors [5]. However, it is possible that other factors such as obesity, alcohol consumption, and diabetes can lead to this condition. Remnant cholesterol, which is highly correlated with triglyceride (TG) levels, is an emerging risk factor for ischemic heart disease [6, 7]. TGs act on blood vessels and cause damage to endothelial cells and vascular smooth muscle cells. It is also known to form foam cells, increase oxidative stress, and impair blood vessel healing [8]. In cases of hypertriglyceridemia, it causes coronary arterial disease, and in serious cases it can lead to pancreatitis and xanthoma, thus becoming a condition for treatment. More recently, studies have used hyperlipidemia as a prognostic factor for diagnosing ischemic heart diseases or cerebrovascular diseases [9, 10]. When hyperlipidemia is diagnosed, improvements to lifestyle and maintenance of appropriate body weight through the control of diet and exercise, as well as the administration of drugs, becomes central to treatment [11]. However, it is considered that common hyperlipidemia (with the exclusion of familial hyperlipidemia) exhibits combined risk factors, involving both genetic and non-genetic components.

The Korea Centers for Disease Control and Prevention and the Genomics Center of the Korea National Institute of Health are conducting the Korean Genome and Epidemiology Study to determine the environmental and genetic risk factors for chronic diseases in Korea with the construction of a prospective large-population cohort study [12]. Among these, from 2005, sets of adult twins and triplets over the age of 35 and their respective family members were recruited for a twin cohort study. Epidemiological data were taken from a questionnaire from body measurement and blood and urine tests [13]. Twin cohort studies observe shared genetic traits where behaviors and disease conditions may differ. This information may find a use in study of disease risk factors arising from complex environmental factors and in work on treatment and prevention of disease [14]. Twin studies using monozygotic (MZ) and dizygotic (DZ) twin pairs who are discordant in their matched pairs for serum lipid level could prove productive assessments of the contributions of lifestyle to hypertriglyceridemia risk with control for genetic and shared environmental factors.

Our study examines TG levels to determine how far they are regulated by lifestyle factors and genetic factors. The use of twins will allow an additional measure of health significance; the findings from our co-twin control study, particularly with reference to MZ twins, will allow non-genetic associations and the effect sizes of the associations to be understood. The effect size of a MZ co-twin control study can be interpreted as illustrating the changes in a single individual that would occur with modifications to a given risk factor. We focused on non-genetic risk factors for TG levels and the quantitative benefits that might be achieved by modifying risk factors. We quantified the influence of factors the contribute to elevated TG levels.

## Methods

### Study subjects

**Fig. 1:**
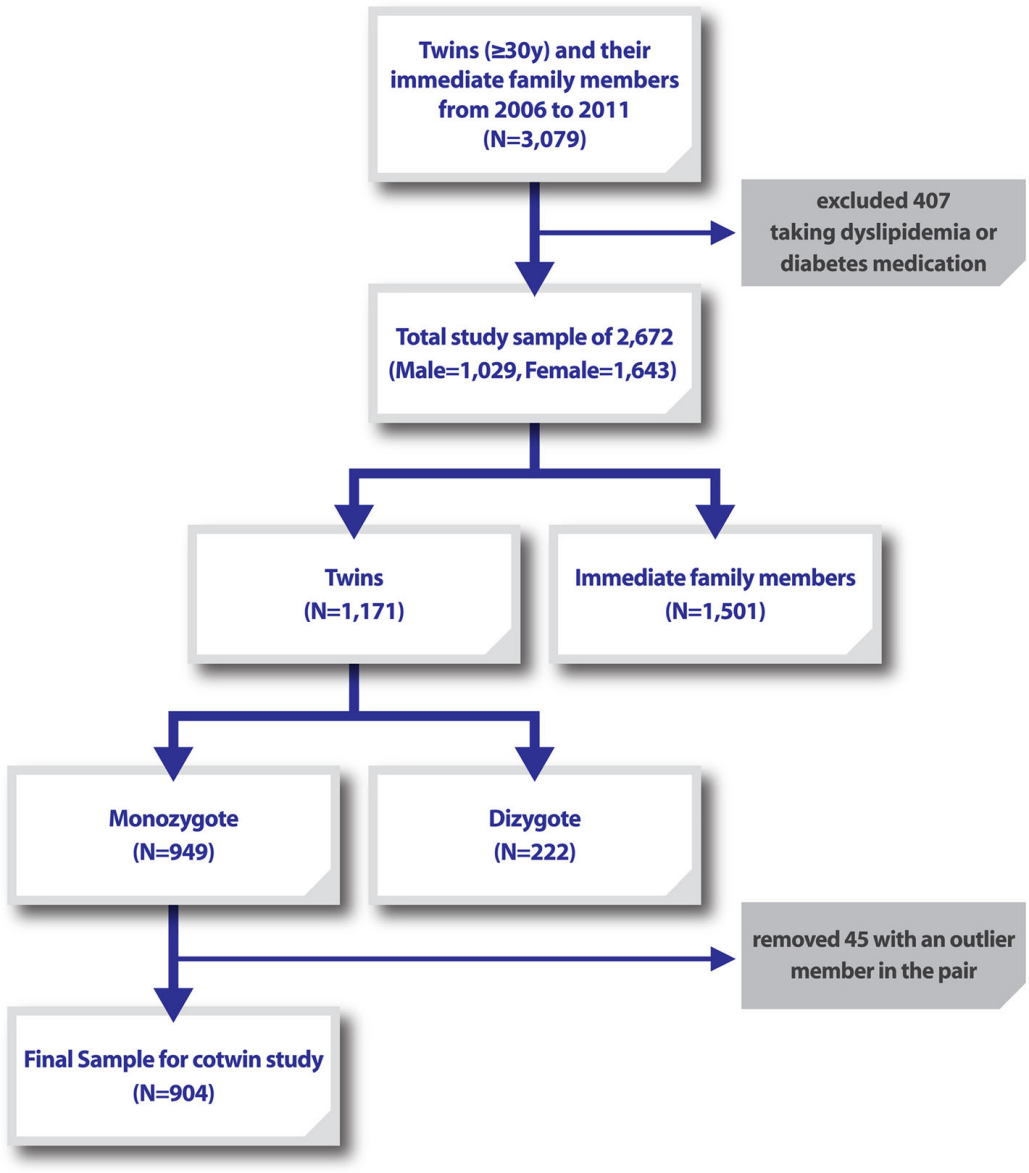
Flowchart of study participation

This study formed part of the Healthy Twin Study, which was created to investigate the complex diseases of adult same-sex twins aged ≥30 years and their family members. The study population include includes 2672 subjects (1029 males and 1643 females), consisting of 949 MZ twins and triplets in sets, 222 DZ twins, and their 1501 parents and siblings. The study was conducted in Seoul, Busan, and Cheonan, Korea, from July 1, 2005, to April 30, 2006 [13]. The exclusion criterion was treatment with dyslipidemia or diabetes medication (Fig. 1). All subjects received a health examination, clinical tests, and physical measurements. All were matched sets of identical twins and triplets. The zygosity of twin and triplet sets was confirmed by the analysis of 16 short tandem repeat (STR) markers (15 autosomal STR markers and 1 sex-determining marker) in 67% of twin or triplet sets. For sets where the results were uncertain, zygosity was confirmed using a questionnaire that was validated through an STR marker study [15]. Written informed consent was obtained from all participants. The study protocol was approved by the ethics committee of Samsung Medical Center and Busan Paik Hospital.

### Anthropometric measurements and questionnaire

This was a multicenter-based study. Every participant was provided with a health examination at one of three clinical centers in different geographical areas (Seoul, Pusan, and Cheonan). Before the work began, the survey methods were standardized between the three centers. A standard protocol was developed and the training of research coordinators and research assistants was regularized. A wide items were assessed in the examination, in the measurement of physical characteristics, the clinical tests performed, and the assessment questions asked (see Table 1 for details). The test batteries were selected from existing, standardized Korean-language versions, or they were freshly adapted for the study through translation and back-translation [13].

**Table 1:** Basic Characteristics of Study Samples

*N* = 2672				
	Male (*n* = 1029)		Female (*n* = 1643)	
	Mean	S.D.	Mean	S.D.
Age (year)	43.9	13.6	43.3	12.7
Fasting glucose (mg/dL)	95.0	14.8	89.7	11.5
Total cholesterol (mg/dL)	190.9	34.5	188.6	36.1
triglyceride (mg/dL)	141.6	107.7	97.2	61.0
<150	704		1401	
150–199	130		131	
200–499	185		110	
>500	10		1	
HDL cholesterol (mg/dL)	46.2	11.2	52.3	12.7
LDL cholesterol (mg/dL)	112.9	30.5	108.8	30.4
Height (cm)	169.7	6.3	156.9	5.7
Weight (kg)	70.4	10.7	56.7	8.2
Waist circumference (cm)	84.7	8.0	77.4	8.7
Hip circumference (cm)	96.1	5.8	93.4	5.6
Body mass index (BMI)	24.4	3.1	23.0	3.2
Body fat (%)	19.9	8.6	27.7	11.1
Alcohol consumption (g/week)	177.6	324.8	46.1	91.4
Smoking (pack-year)	13.5	17.0	0.4	2.3
Dietary intake				
Total energy (kcal)	1942.7	791.2	1776.9	861.4
Protein (g)	67.1	32.8	62.5	41.1
Fat (g)	37.4	23.7	33.5	29.3
Carbohydrate (g)	328.8	134.4	303.4	136.3
Cholesterol (mg)	203.2	154.7	193.0	194.9
MET^a^	7096.1	11,701.4	5861.0	9675.7

Abbreviation: *SD* Standard deviation

^a^metabolic equivalent of task using Physical Activity Score

Anthropometric measurements of subjects wearing light clothing and no shoes were conducted by experienced research staff. Height was measured to the nearest 0.1 cm with a standiometer (BSM 330, Biospace Co., Ltd., Seoul, South Korea). Body weight was measured to the nearest 0.1 kg with an In-Body machine (InBody 520, Biospace Co., Ltd., Seoul, Korea). body mass index (BMI, kg/m^2^) was calculated as weight (kg) divided by height squared (m^2^). The World Health Organization (WHO) Regional Office for the Asia Pacific Region recommends that obesity in Asian populations be defined as BMI ≥25 kg/m^2^. The Korean Society for the Study of Obesity also investigated BMI cutoff figures for obesity-related disease and adopted the definition of the Regional Office of the WHO. Korean government organizations also use this definition officially in their definition and implementation health policies regarding obesity in Korea [16]. Waist circumference was measured around the midpoint between the top of the iliac crest and the lowest rib while nude. Blood pressure was taken when participants had been at rest for more than 5 min, using an automatic blood pressure measurement system (CK-301, Spirit Medical Co., Taiwan). The second measured value was used to determine blood pressure for above-normal range. When both the preceding measurements were within the normal range, another measurement was manually conducted.

Demographic information affecting TG levels, such as age, sex, alcohol intake, smoking habits, and physical activity, was determined. Past and current medical history, family history of disease, and food frequency were also surveyed. Alcohol intake was calculated as alcohol consumption over the previous year (g/year). Smoking was assessed as smoking consumption over the previous year (packs/year). Smoking behavior was classified into the three groups non-smokers, ex-smokers, and current smokers, depending on current smoking status. Physical activity was classified into the three groups highly active (1500 MET-minute/week for any strenuous physical activity practiced more than 3 days a week or 3000 MET-minute/week for any activity conducted daily), moderately active (intense physical activity for more than 20 min per week or moderate activity or walking for more than 30 min 5 days a week), and no physical activity/inactive (below a moderate level of physical activity).

### Clinical examination: blood tests

Blood samples were drawn after a minimum 12-h overnight fast, collected in EDTA-containing tubes, and centrifuged at 3000 rpm for 20 min at 4 °C (Hanil Science Industrial Co., Ltd., Seoul, Korea). Fasting plasma levels of glucose, TG levels, total cholesterol, and HDL cholesterol were assessed with an autoanalyzer (Cobas 6000, Roche Diagnostics International Ltd., Rotkreuz, Switzerland). LDL cholesterol levels were calculated with the following equation, used by both Friedwald [17] and Lauer [18]:

$$ \mathrm{LDL}\ \mathrm{cholesterol}=\mathrm{total}\ \mathrm{cholesterol}-\mathrm{HDL}\ \mathrm{cholesterol}-\left(\mathrm{TG}\ \mathrm{level}/5\right). $$

### Statistical analysis

The mean values of the measured variables were compared between male and female twins using *t* tests. The correlations between the measured variables from within-pair difference methods were analyzed using Pearson correlation.

The rationale for the twin study is that MZ twins and triplets share 100% of their genetics, so the differences between members of a sets are due only to the environment [19]. This MZ co-twin–control design in its very nature matches for age, sex, and genetic factors. Thus, the analysis of the within-set differences in TG levels as a linear function of within-set differences in alcohol consumption, intake of dietary carbohydrates, and BMI, researchers can control for common genetic factors.

A total population of 2672 individuals was subdivided into two groups by sex, and a basic comparison was made. Normal distribution was determined by univariate analysis; in cases where a bias appeared in TG scores, a log scale was used to indicate a geometrical average, and other measurement scores were expressed as means with standard deviations. Age, BMI, alcohol consumption, smoking habits, and fasting blood glucose variables were categorized, and differences in the TG levels were determined. Furthermore, the effects of each variable on TG levels were determined by regression analysis [20]. From these, to determine the factors the lead to changes in blood TG levels, a mixed linear model was utilized for the multivariate analysis. The correlation structures resulting from familial factors were adjusted using the mixed linear model; in particular, the effects of the familial factors and twins were adjusted with consideration for random effects. For the identical twins, the differences in the consecutive variables for a population of 452 sets were calculated, and a co-twin study was conducted so that factors previously identified as playing an important role on the increasing TG levels could be entirely accounted for by non-genetic factors [19].

All statistical analyses were performed using SAS 9.4 (SAS Inc., Cary, NC, USA). The significance level was set at *p* < 0.05.

## Results

### Subject characteristics

In all, the population for this study totaled 2672, 1029 men and 1643 women, average ages of 43.9 ± 13.6 and 43.3 ± 12.7 years, respectively. Mean blood TG concentration was significantly higher for men, at 141.6 ± 107.7, than for women, at 97.2 ± 61.0. Mean total cholesterol levels were not significantly different, with an average of 190.9 ± 34.5 for men and 188.6 ± 36.1 for women; mean waist measurements were 84.7 ± 8.0 for men and 77.4 ± 8.7 cm for women; mean body weights were 70.4 ± 10.7 and 56.7 ± 8.2 kg, respectively; and mean BMIs were 24.4 ± 3.1 and 23.1 ± 3.2 kg/m^2^, respectively. Measures of lifestyle factors follow: 866 self-reported as non-drinkers and 1806 as drinkers, and there were 1758 non-smokers, 352 past smokers, 521 current smokers, and 41 non-classified. The mean weekly alcohol consumption (g) of currently smoking drinkers was different from the rest of the population, with 177.6 ± 324.8 g for men and 46.1 ± 91.4 g for women, while smoking, calculated on a pack-year basis for this group was quite different between the sexes, with 13.5 ± 17.0 for men and 0.4 ± 2.3 for women. A comparison of food intake showed a significant difference between sex as well, with 1942.7 ± 791.2 for men and 1776.9 ± 861.4 daily kcal for women. However, no statistical difference was found in proportions of protein, fat, carbohydrate, and cholesterol intake adjusted for total diet (Table 1).

To determine the correlation between the variables, the results were graphed, and linearity was only visually detectable for cholesterol and LDL cholesterol and the group of measurements for waist and hip, body weight, and BMI. There was a clear linearity between total food intake and the protein, fat, carbohydrate, and cholesterol intakes. Similarly, a major factor that determines blood TG levels was LDL or HDL cholesterol, and another key factor was BMI, which takes into account both height and weight.

### TG levels and other factors

TG concentrations and factors that influence TG levels were categorized to determine the correlations. TG concentrations (mg/dL) lower than 150 were categorized as low, 150–199 as normal, 200–499 as slightly high, and 500 and up as very high. On this classification, 68.4% (704) of men and 85.3% (1401) of women were in the low class, 130 men and 131 women were in the normal category, and there were 185 men and 110 women in the slightly high range.

TG levels were found to be greater with greater BMI scores, irrespective of sex. Moreover, 60% of the total population in the study was in the 30–40 year category, and a significant increase in TG levels was observed with increasing age, although not in men. The normal range for fasting glucose is below 100 mg/dL, and 80% of the values of the total population in the study were clustered in that range. This largest group was thus subcategorized into the below-90 and 90–100 mg/dL groups. Analysis showed that for higher fasting glucose levels, there was a clear increase in TG levels for both men and women. The average recommended level of daily alcohol consumption is 20 g, and the participants were divided into consumption groups of 60 and 160 g. It has been reported that for daily alcohol consumption of 160 g per day or more, a greater than 10-fold increase in the risk for liver cirrhosis appears. This study investigated average weekly alcohol consumption; it was not possible to determine the risk groups. For men, with increases in alcohol consumption increases, it was evident that the TG levels were significantly increased; however, in women, TG levels were greatest among non-drinkers, and even where alcohol consumption was increased, neither the TG level nor the trend was observed (Table 2).

**Table 2:** Factors Associated with Serum Triglyceride Level Using Stratification

	Male (*n* = 1029)		Female (*n* = 1643)	
	No. (%)	TG level	No. (%)	TG level
BMI				
< 20(Underweight)	63 (6.12)	91.8	264 (16.07)	73.8
20–23 (Standard weight)	263 (25.56)	109.4	633 (38.53)	86.5
23–25 (Overweight)	288 (27.99)	141.4	352 (21.42)	100.9
25–30 (Obesity)	382 (37.12)	165.3	346 (21.06)	126.7
> 30 (Severe obesity)	33 (3.21)	220.5	48 (2.92)	127.3
Age				
-29	127 (12.34)	143	152 (9.25)	78.1
30–39	360 (34.99)	147.5	633 (38.53)	80.7
40–49	212 (20.60)	140.6	380 (23.13)	89.4
50–59	154 (14.97)	143.5	250 (15.22)	127.8
60-	176 (17.10)	128.1	228 (13.88)	135.3
FBS (mg/dL)				
< 90	352 (34.21)	84.4	905 (55.08)	83.2
90–100	439 (42.66)	93.9	559 (34.02)	93.6
100–125	211 (20.51)	106.8	163 (9.92)	105.6
> 126	27 (2.62)	159	16 (0.97)	159.6
Alcohol consumption (g/week)				
0	182 (17.69)	111.6	684 (41.63)	106.0
< 20	127 (12.34)	135.3	516 (31.41)	92.4
20–60	188 (18.27)	130.9	256 (15.58)	86.0
0–160	237 (23.03)	141.3	115 (7.00)	91.4
> 160	295 (28.67)	170.0	72 (4.38)	97.3

To determine the factors that are linked to blood TG concentrations, linear regression modeling analysis was performed, with normalized age and gender. Among blood measurements, fasting glucose, total cholesterol, HDL cholesterol, and LDL cholesterol levels showed a statistically significant relationship with TG concentrations; in particular, HDL cholesterol levels showed a negative correlation. Weight, waist and hip measurements, distribution of fat, BMI, and obesity showed a significant correlation, but height did not. Finally, weekly alcohol consumption showed a significant correlation, but there was no positive correlation with food intake or cigarette smoking (Table 3).

**Table 3:** Correlation between Serum Triglyceride and Each Variable Using Simple Linear Regression Analysis (sex, age adjusted)

Variables	Coefficient	95% confidential interval	
Fasting glucose (mg/dL)	1.094	0.849	1.339
Total cholesterol (mg/dL)	0.549	0.462	0.636
HDL cholesterol (mg/dL)	−2.036	−2.279	−1.793
LDL cholesterol (mg/dL)	0.145	0.403	0.250
Height (cm)	0.551	−0.015	1.118
Weight (kg)	2.262	1.940	2.585
Waist circumference (cm)	2.990	2.630	3.370
Hip circumference (cm)	2.607	2.072	3.143
Body mass index (BMI)	6.813	5.853	7.774
Body fat (%)	1.197	0.894	1.501
Dietary intake			
Total energy (kcal)	0.003	−0.000	0.007
Protein (g)	0.060	−0.022	0.141
Fat (g)	0.032	−0.083	0.147
Carbohydrate (g)	0.023	−0.000	0.045
Cholesterol (mg)	−0.001	− 0.018	0.017
MET	−0.000	−0.001	− 0.000
Alcohol consumption(g/week)	0.034	0.018	0.049
Smoking (pack-year)	0.106	−0.200	0.415

After normalizing the data to age and gender, factors previously related to TG levels, such as fasting levels of glucose, HDL, LDL, body weight, waist and hip measurement, body fat index, and alcohol consumption were subjected to a multivariate analysis; it was found that fasting glucose and HDL cholesterol significantly affect blood TG levels, and BMI, which is an indicator of body shape, and alcohol consumption also have significant effects (Table 4).

**Table 4:** Factors Associated with Serum Triglyceride Level Using Multiple Linear Regression Analysis (age, sex adjusted)

Variables	Coefficient	95% Confidential Interval	
Sex*	−17.508	−23.956	−11.059
Age (year)	0.211	−0.023	0.445
Fasting glucose (mg/dL)*	0.839	0.606	1.072
HDL cholesterol (mg/dL)*	−1.835	−2.080	− 1.589
LDL cholesterol (mg/dL)	0.080	−0.020	0.180
Alcohol consumption (g/week)*	0.040	0.025	0.055
Body mass index (BMI)*	4.385	3.406	5.363

*p value< 0.01

The TG levels were considered a resulting variable, so to determine causative factors, an analysis was performed with same-family subjects to indicate random effects. Because such analysis could cause high multicollinearity, total, HDL, and LDL cholesterol were excluded from the analysis. Waist and hip measurement and BMI showed a significant correlation that could be explained with one major variable; therefore, BMI alone was selected for comparison by multivariate analysis. After other variabilities were normalized, men were found to have higher TG levels than women, and BMI also showed significant correlation. Moreover, it also showed strong correlation with AUDIT, which is an indicator of alcohol dependency. In the case of smoking and alcohol consumption, TG levels appeared to decrease going from non-smoker to smoker, however this difference was not statistically significant (Table 5).

**Table 5:** Factors Associated with Serum Triglyceride Level Using Multiple Linear Regression Analysis (considering random effect about the family)

	Estimate	Standard error	T value	*P* value
Age	0.08	0.39	0.21	0.83
Sex (Male)*	31.96	7.02	4.55	<0.01
Sex (Female)	0	.	.	.
BMI*	6.91	0.89	7.79	<0.01
Dietary intake				
Total energy (kcal)	−0.02	0.1	−0.18	0.86
Protein (g)	0.49	0.44	1.12	0.26
Fat (g)	−0.41	0.89	−0.46	0.65
Carbohydrate (g)	0.06	0.4	0.15	0.88
SSCholesterol (mg)	−0.01	0.03	−0.33	0.74
Physical Activity	0	0	−1.91	0.06
No drinking	−16.64	8.71	−1.91	0.06
Past drinker	−12.1	8.73	−1.39	0.17
Current drinker	0	.	.	.
Alcohol dependency (AUDIT)*	0.97	0.45	2.16	<0.05
Non-smoker	13.6	20.09	0.68	0.5
Past smoker	−0.1	8.15	−0.01	0.99
Current smoker	0	.	.	.
Smoking (pack-year)	0.3	0.42	0.7	0.48

*p value <0.05

### Co-twin control analysis

Out of a total of 949 MZ twins and triplets in sets, to create a co-twin control, the 45 pairs in which one member was an outlier were removed, and data from the remaining 904 were analyzed. The variable difference was calculated from the 452 twins and triplets and correlations in their data were compared. The factors that showed difference in the blood TG levels between the twins with the same genetic material were as follows: fasting glucose, HDL, LDL, BMI, and alcohol. Alcohol consumption was excluded. As a result, age, fasting blood glucose, HDL cholesterol, and BMI showed significant correlation. When non-value measurements were excluded but alcohol consumption was considered, age, HDL, LDL, BMI, and alcohol consumption all showed significant correlations (Table 6).

**Table 6:** Factors Associated with Serum Triglyceride Level in the Monozygote subgroup (co-twin control study)

Variables	Coefficient	95% Confidential Interval	
Age (year)*	1.67	0.06	3.28
Sex	20.53	−2.69	43.76
Fasting glucose (mg/dL)	0.75	−0.57	2.07
HDL cholesterol (mg/dL)*	−1.26	− 2.47	−0.06
LDL cholesterol (mg/dL)*	−0.56	−1.10	−0.01
BMI**	9.94	3.42	16.46
Body fat (%)	1.13	−1.12	3.38
Dietary intake of Carbohydrate (g)	0.04	−0.04	0.12
Alcohol consumption (g/week)*	0.08	0.02	0.14

*p value <0.05

**p value <0.01

## Discussion

By comparing adjusted TG levels between MZ twins who share genome types and developmental backgrounds, we were able to infer the degree of contribution of non-genetic factors to the TG levels.

It is not yet clear whether hypertriglyceridemia is an independent risk factor for coronary heart disease. However, observational studies that increased cardiovascular risk is associated with hypertriglyceridemia [21]. In addition, metabolic syndrome includes hypertriglyceridemia and low HDL cholesterol levels [22]. Studies of lipid-lowering therapies found that atherogenic dyslipidemia patients had reduced coronary risk [23, 24].

Diagnosis of hypertriglyceridemia indicates lifestyle modification, including smoking cessation, moderate alcohol consumption, and smaller intake of food containing high carbohydrates; increased physical activity and reduction in obesity should be pursued before pharmaceutical intervention. The reason for being cautious of high hyperglycemia as a result of carbohydrate intake is that high levels of glucose intake can lead to rising TG levels; this is also supported by studies finding that lowering the blood HDL has a deleterious effect on lipid and glucose metabolism, as in the case of hyperinsulinism [25, 26]. Moreover, low levels of HDL cholesterol combined with high TG levels can act as a major causative factor in the development of ischemic heart diseases [27]. Further, obesity can be considered to be a risk factor for the development of hypertriglyceridemia and waist measurement or BMI can be used to indicate obesity quantitatively and it is already known that as the levels increase, blood TG concentrations rise, the dangers of developing coronary diseases consequently increase [28–30].

The analysis makes clear that within the twin cohort, the risk factors that influence the hypertriglyceridemia concentrations in the population were similar to previous findings: high fasting blood glucose, total cholesterol level, low HDL cholesterol, high LDL cholesterol, high obesity measurements (weight and waist measurements), alcohol consumption, and carbohydrate-based diet have significant correlation with hypertriglyceridemia. Lifestyle factors such as smoking and exercise were found to have a close relationship with TG concentrations in the previous studies [31, 32]. However, this finding has persistently been considered controversial by authors, and this study failed to find a significant correlation. It must also be considered that there were few smokers in this study, meaning that analysis of related results were weakened, and the answers derived from the physical activity questionnaire were difficult to understand. In fact, taking into account that in the current study, 543 individuals gave no-value answers, it is difficult to extrapolate a relationship of significance between physical activity level and hypertriglyceridemia.

A previous study showed a significant correlation with carbohydrate intake in particular; however, this study failed to confirm this. Previous studies have shown that TG levels have a significant inverse correlations with protein, dietary fiber, polyunsaturated fat, and positive correlations with saturated fat [33].

The limitation of the current study include its dependence on the recall method used to obtain nutritional information, which may not represent actual dietary patterns, and that it did not perform a further analysis after categorizing lipids into saturated and unsaturated fats.

An index to represent obesity using waist and hip measurements, body weight, BMI, and fat distribution was analyzed using pair-wise correlation, and the first four, factors excluding fat distribution, showed almost identical explanatory power. Thus, only one factor of these four was chosen, leading to almost identical results. If blood triglyceride concentration is considered a resulting variable, multivariate analysis can be performed using a random effect model to determine what factors influence it [20]. It was assumed that independent variable would have a random distribution; in this study, the familial relationship was considered to provide a random effect for the performance of rarity analysis [34, 35]. The results indicated that when other variabilities were normalized, men had significantly higher levels of TG and values of BMI than women. Furthermore, AUDIT, which represents alcohol dependency, exhibited a significant correlation. Although TG levels decrease as levels of alcohol consumption fall, this trend was not statistically significant. Smoking also showed a similar trend, in that non-smokers showed lower TG levels than smokers, but this difference was not significant.

## Conclusions

This current study identified adjustable factors from among those that can explain the difference in TG levels; further, it was found possible to predict how far TG levels could be reduced. For example, the fasting blood glucose level of the upper 90% of the population falls in the glucose intolerant range of 103 mg/dL and it is expected that TG levels can be reduced to about 20.2 mg/dL if fasting blood glucose is reduced to 80 mg/dL, which would match the levels of the upper 10% of the population. BMI, which is a ratio of height and weight, changes depending on the variable, showed a correlation coefficient in the range of 7.8–9.9, which would predict that a TG level of 7.8–9.9 mg/dL could be lowered for any BMI reduction. Even where differences in height are considered, when body weight is reduced by about 3–4 kg, it is expected that a TG level of 8–10 mg/dL can be lowered. The BMI of the upper 10% of the population by body weight in the current study was 27.6, and that of the lower 10% was 19.7, and if normal body weight can be achieved from the obese group by adjusting lifestyle, TG levels of greater than 35 mg/dL can be lowered without administering pharmaceutical agents. In the same way, reducing alcohol consumption by 50 g/week (about two glasses of beer) might reduce TG levels by 4.0 mg/dL. Although significant results were not confirmed in the confidence interval, reducing dietary carbohydrate intake by 100 g/day (about a bowl of rice) could be expected to decrease TG levels by 4.0 mg/dL. This provides important evidence for the therapy of metabolic diseases.

## Data Availability

The data used in this paper were provided by the Korean Center for Disease Control Research Program contract. We signed a legally binding agreement with the Center that we would not share the original data to any third parties. Although interested researchers can request data using the telephone number + 82–43–719-6720, the Korean Center for Disease Control has final say on distribution of the data.
